# Isolating climatic, tectonic, and lithologic controls on mountain landscape evolution

**DOI:** 10.1126/sciadv.add8915

**Published:** 2023-01-20

**Authors:** Joel S. Leonard, Kelin X. Whipple, Arjun M. Heimsath

**Affiliations:** School of Earth and Space Exploration, Arizona State University, Tempe, AZ, USA.

## Abstract

Establishing that climate exerts an important general influence on topography in tectonically active settings has proven an elusive goal. Here, we show that climates ranging from arid to humid consistently influence fluvial erosional efficiency and thus topography, and this effect is captured by a simple metric that combines channel steepness and mean annual rainfall, *k_snQ_*. Accounting for spatial rainfall variability additionally increases the sensitivity of channel steepness to lithologic and tectonic controls on topography, enhancing predictions of erosion and rock uplift rates, and supports the common assumption of a reference concavity near 0.5. In contrast, the standard channel steepness metric, *k_sn_*, intrinsically assumes that climate is uniform. Consequently, its use where rainfall varies spatially undermines efforts to distinguish climate from tectonic and lithologic effects, can bias reference concavity estimates, and may ultimately lead to false impressions about rock uplift patterns and other environmental influences. Capturing climate is therefore a precondition to understanding mountain landscape evolution.

## Teaser

Accounting for climate unlocks potential to disentangle primary factors controlling the evolution of mountain topography.

## INTRODUCTION

Covariation among climate, tectonics, and lithology is common in mountain landscapes and has long confounded attempts to isolate and quantify their respective roles in moderating topography and erosion (*1*–*3*). Climate’s role, in particular, has been the subject of vigorous debate, and clearly determining whether climate importantly influences topography remains a critical challenge (*1*, *4*–*8*). In principle, the normalized channel steepness index (*k_sn_*), a widely used metric that has produced useful correlations with erosion on millennial time scales in diverse settings, has the potential to detect such an influence (*1*–*3*). However, a key limitation of *k_sn_* is its reliance on upstream drainage area as a discharge proxy, thus building in an assumption of spatially uniform rainfall.

A promising alternative to *k_sn_* is the variant, *k_snQ_* (*1*)

$$k_{snQ}={SQ}^{\theta_{\mathrm{ref}}}$$ (1)

where *S* is the river slope, *Q* is the water discharge, and θ_ref_ is the reference concavity index. *k_snQ_* is a generalized version of similar metrics [e.g., (*9*, *10*)] that uses the product of drainage area (*A*) and average upstream rainfall ($\bar{P}$) estimated from mean annual rainfall (MAP) as an improved discharge proxy (*1*). This allows *k_snQ_* to account for spatial rainfall variability (*11*). Although the stochastic natures of storms and floods are not captured, MAP resolves spatial patterns well and scales quasi-linearly with larger geomorphically relevant discharges (*12*). To the extent MAP captures the principal influence of climate, its near-global coverage and relative simplicity offer notable advantages over reliance on sparse stream gauges. A critical test to evaluate the usefulness of incorporating MAP is by comparing the capacities of *k_sn_* and *k_snQ_* to predict erosion rates where rainfall is spatially variable.

**Fig. 1.**
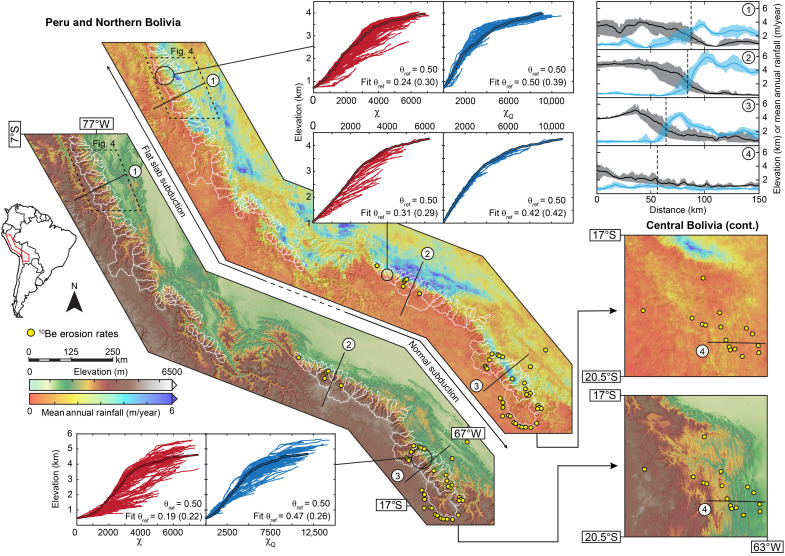
Map synthesizing topography, rainfall, subduction regime, and channel steepness patterns from the north-central Andes of Peru and Bolivia. Topographic analysis based on catchments outlined in white. Locations of ^10^Be erosion rate measurements used for this study are shown with yellow circles (54 in total). Comparisons between χ-*z* (red) and χ*_Q_*-*z* (blue) profiles for representative catchments along strike. Trunk profiles are highlighted in black. All panels are plotted with θ_ref_ = 0.5 and show optimized θ_ref_ values determined using stream networks with a minimum upstream drainage area of 5 km^2^ (and 1 km^2^); see the main text for details. Numbered lines 1 to 4 on maps show centerline locations of 50-km-wide swath profiles of topography and rainfall, depicted on the top right. Swaths show mean, minimum, and maximum values; vertical dashed lines show the position where mean elevation reaches 2 km; see the main text for details.

Here, we present a detailed, large-scale analysis of topography as represented in river profiles and ^10^Be erosion rates from the north-central Andes of Peru and Bolivia (Fig. 1), testing the hypothesis that *k_snQ_* is a better predictor of erosion rates than *k_sn_* (*1*, *11*). In doing so, we highlight assumptions implicit to each metric and quantitatively evaluate the implications of carrying forward these different sets of assumptions on understanding the roles of climate, tectonics, and lithology on landscape evolution. This landscape is characterized by a ~20- to 50-km-wide band of high topographic relief and experiences a marked regional, but locally variable, orographic rainfall gradient that spans much of the global range of mean annual rainfall. High elevations typically receive approximately <0.25 m/year, while lower elevations experience ~3 to 6 m/year of rainfall. The study area spans >1500 km along strike and, in addition to extreme climate variations, encompasses diverse tectonics (e.g., variable subduction geometry and seismicity) (*13*, *14*) and lithology (*15*, *16*). This degree of complexity is common in tectonically active ranges and is ideal for comparing the abilities of *k_sn_* and *k_snQ_* to extract meaningful information about primary drivers of landscape evolution.

### River incision theory and metrics

A common framework for interpreting *k_sn_* and *k_snQ_* is the stream power model (SPM). The SPM can be cast in terms of drainage area (*A*) or discharge (*Q*)

$$E=KA^{m}S^{n}$$ (2a)

$$E=K_{p}Q^{m}S^{n}$$ (2b)

$$K=K_{p}{\bar{P}}^{m}$$ (2c)

where *E* is the erosion rate [equal to the uplift rate (*U*) at steady state], *K* and *K_p_* are erosional efficiency coefficients, and *m* and *n* are positive constant exponents that depend on erosional process mechanics and runoff variability (*1*, *2*, *17*). Dependence on runoff variability and erosional thresholds more broadly suggests that *n* ought to vary with climate (*2*, *12*, *18*–*23*). However, the primary influence of climate is encapsulated within *K*, along with a myriad of other factors (e.g., rock properties). By contrast, *K_p_* is, in principle, independent from rainfall and, to the extent that rainfall is the primary relevant component, climate (*1*, *11*). Effects of other factors such as rock properties, weathering, sediment flux and size, and vegetation that do not scale with rainfall are still subsumed in *K_p_* (*17*, *24*–*28*).

Interpreting channel steepness patterns requires knowledge of the reference concavity index, θ_ref_, which describes the scaling between discharge and channel slope and allows comparisons between rivers of different sizes (*29*). Values near 0.5 are expected from theory and under uniform conditions θ_ref_ = *m*/*n* (*2*, *30*, *31*). However, spatially and/or temporally variable conditions affect profile concavity and channel steepness (*11*, *32*–*35*).

River profile collinearity in χ-elevation plots (χ-*z*) has become a popular method to determine optimal θ_ref_ values from topography (*34*, *36*, *37*). This method leverages the SPM prediction that, provided θ_ref_ is set appropriately, the χ-transform linearizes river profiles adjusted to uniform conditions with slopes equal to *k_sn_* (*34*). Assuming that rivers of varying sizes are adjusted to uniform conditions, the optimal θ_ref_ value that best approximates *m*/*n* is that which minimizes differences among profile slopes (*34*, *36*, *38*, *39*). Collinearity can be quantified by comparing *k_sn_* values and optimized by minimizing variability (disorder) in χ-*z* (*36*, *38*, *39*). However, where either rainfall or rock erodibility varies spatially (a common condition), the θ_ref_ value that best achieves collinearity among profiles may not be meaningful.

We can evaluate the effect of rainfall on collinearity by modifying χ to scale with *Q* (i.e., $\bar{P}$*A*), which we define as χ*_Q_* (see Materials and Methods) (*11*, *34*, *40*). The slope of river profiles on χ*_Q_*-*z* plots is *k_snQ_*. Therefore, spatial variations in rainfall that drive differences in channel steepness should not affect collinearity in χ*_Q_*-*z*, improving θ_ref_ estimates provided that catchments are adjusted to the rainfall pattern.

## RESULTS AND DISCUSSION

### River profile analysis

Transversely draining (trunk) rivers across our study area exhibit a consistent, distinctive pattern of higher *k_sn_* than their tributaries using a typical θ_ref_ = 0.5, shown in χ-*z* plots (Fig. 1). As outlined above, the simplest interpretation of this pattern is that rivers are adjusted to spatially variable conditions (*U* or *K*), implying that trunk and tributary rivers systematically experience different conditions (e.g., average rainfall) (*11*). Here, the discrepancy between trunk and tributary channel steepness is greatly reduced in χ*_Q_*-*z* space using θ_ref_ = 0.5 (Fig. 1), bolstering the notion that profiles here are importantly influenced by spatially varying rainfall and that *k_snQ_* captures this influence. Alternatively, the discrepancy between large trunk and small tributary rivers may simply reflect an incorrect choice of θ_ref_, which must be evaluated.

**Fig. 2.**
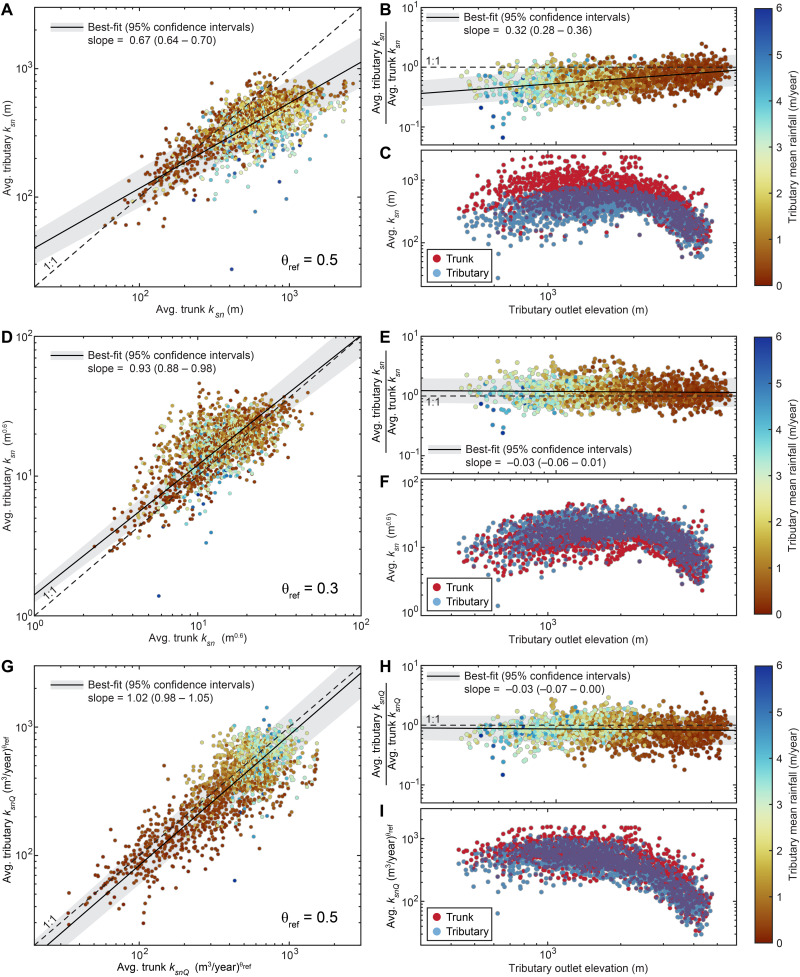
Regional analysis of relationships among trunk and tributary channel steepness patterns and rainfall. Comparison between trunk and tributary *k_sn_* for θ_ref_ = 0.5 (**A** to **C**), θ_ref_ = 0.3 (**D** to **F**), and *k_snQ_* (θ_ref_ = 0.5) (**G** to **I**). Dashed lines show 1:1 relationship. Solid black lines show total least-squares (A, D, and G) and ordinary least-squares (B, E, and H) regressions with 95% confidence intervals in gray. Spatial pattern of erosion predicted by trunk and tributary rivers shown in (C), (F), and (I).

Quantitative comparisons between tributary *k_sn_* or *k_snQ_* and equivalent trunk river segments (1694 tributary-trunk pairs) across the study area are shown in Fig. 2. This confirms that trunk *k_sn_* is higher than that of tributaries, on average, using a typical θ_ref_ = 0.5, and reveals greater disparities at lower elevations (Fig. 2, A to C). Also, tributaries, which are more sensitive to varying environmental conditions (*11*, *33*), indicate a transition between increasing and decreasing *k_sn_* with outlet elevation at ~2 km. Although this pattern is consistent with rainfall variations (Fig. 1), spatial gradients in uplift rate cannot be discounted without first establishing the sensitivity of *k_sn_* to rainfall.

Alternatively, trunk and tributary *k_sn_* values are nearly equal on average using θ_ref_ = 0.3 (Fig. 2D). If θ_ref_ = 0.3 is a good description of these profiles, then this finding would imply that *K* is approximately uniform and that *K* and, thus, *k_sn_* are not strongly affected by rainfall (Fig. 2, D and E). The *k_sn_*-elevation pattern is not sensitive to θ_ref_ (Fig. 2F), but quasi-uniform *K* would suggest that *k_sn_* variations likely reflect spatial gradients in uplift rate. However, θ_ref_ = 0.3 is low compared to commonly observed values (*31*) and, further, is incompatible with the high concavities observed along trunk rivers in this part of the Andes (*32*, *41*), rendering the appropriateness of the “optimal” θ_ref_ = 0.3 questionable.

In contrast, *k_snQ_* is predicated on the assumption that *K* depends strongly on climate (Eqs. 1 and 2c). The relationship between trunk and tributary *k_snQ_* using θ_ref_ = 0.5 is indistinguishable from linear (Fig. 2, G and H). That variations in *K* expected from discharge accumulation under the observed rainfall pattern reconcile differences between trunk and tributary channel steepness implies that rivers are largely adjusted to the rainfall pattern. This likely precludes recent substantial changes to the rainfall pattern as any such changes would force trunk and tributary *k_snQ_* out of alignment. Moreover, discharge accumulation patterns have spatial complexity unlikely to be mirrored by uplift patterns. The *k_snQ_* pattern is distinct from that of *k_sn_*, suggesting quasi-uniform, high erosion rates in tributaries with outlet elevations of <2 km, above which tributary headwaters often tap the low-relief plateau (Fig. 1), causing catchment mean *k_snQ_* to decline (Fig. 2I).

### Collinearity: Along-strike variations and limitations

**Fig. 3.**
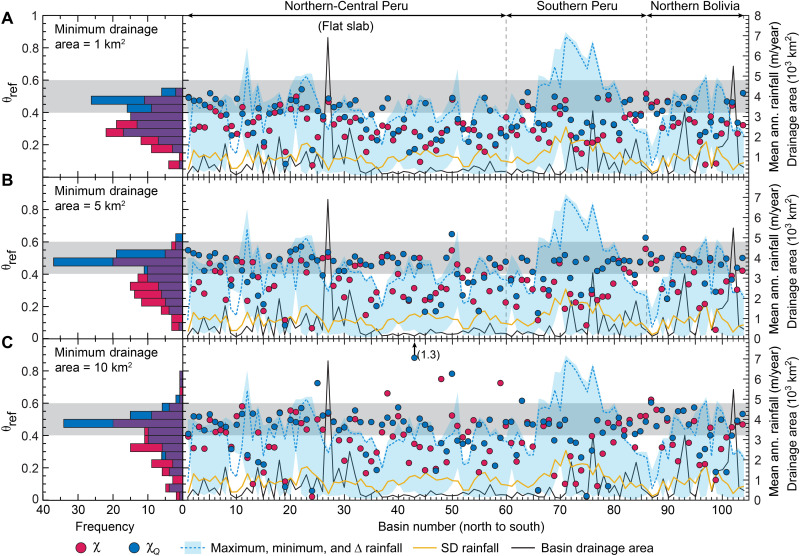
Analysis of optimized θ_ref_ values individually fit to each catchment along strike. Comparisons are shown between stream networks calculated with minimum drainage areas of 1 km^2^ (**A**), 5 km^2^ (**B**), and 10 km^2^ (**C**), for both χ-*z* profiles (red) and χ*_Q_*-*z* (blue). Optimized θ_ref_ values are plotted alongside catchment rainfall characteristics and drainage area. Histogram shows the frequency of optimized values; see Materials and Methods and the Supplementary Materials.

We next explore the potential for along-strike variations in θ_ref_ by optimizing the collinearity of all profiles within each catchment—a more detailed, local analysis than the regional analysis of tributary-trunk pairs above. Optimized θ_ref_ values for χ-transformed stream networks have a central tendency of 0.25 to 0.3, while χ*_Q_*-transformed networks have a bimodal distribution with a stronger mode at 0.45 to 0.5 and a weaker one at 0.25 to 0.3 (Fig. 3A). Although θ_ref_ optimizations in individual catchments are, collectively, consistent with the regional analysis, they suggest substantial intercatchment variability. If true, then this would complicate topographic analyses and potentially confound efforts to evaluate relationships between erosion and topography (*29*, *37*).

Notably, increasing the minimum drainage area in the channel network from 1 to 5 km^2^ markedly reduces variability in optimized θ_ref_ for χ*_Q_*-transformed stream networks (Fig. 3B). With this trimmed stream network, a single mode at 0.45 to 0.5 emerges with ~70% of catchments falling within the typical 0.4 to 0.6 range, reinforcing the interpretation that channels are largely adjusted to the rainfall pattern. Remaining catchments outside this range have variable lithology (*15*, *16*) and/or are small (<200 to 300 km^2^) (see the Supplementary Materials). Increasing this threshold further to 10 km^2^ has little additional effect in our study basins except to increase the frequency of outliers, which reflects a trade-off where the sparsity of tributaries in the remaining stream network becomes a limiting factor (Fig. 3C). Optimized θ_ref_ values based on χ-transformed profiles are less affected. A broad mode centered at 0.3 to 0.35 includes most catchments, but an additional mode at 0.45 to 0.5 appears. Catchments comprising the 0.45 to 0.5 mode tend to have less spatially variable rainfall (lower SD). This shift toward a bimodal distribution continues as we increase the drainage area further, but outliers also become more common, similar to what we observe for χ*_Q_*-transformed stream networks.

We suggest that the effect of increasing the minimum drainage area suggests that variability of optimized θ_ref_ in the χ*_Q_*-*z* analysis (Fig. 3A) largely reflects limitations imposed by the spatial resolution of the rainfall data. At 1 km^2^, the area defining streams is much smaller than the resolution of the rainfall grid (~20 km^2^). Consequently, rainfall estimates at small drainage areas, particularly in high-relief tributaries with associated spatial rainfall variability, are likely to be inaccurate. This can produce anomalous *k_snQ_* values that can bias the results of the θ_ref_ optimization. Smaller catchments comprising fewer streams are more sensitive to anomalous values as these artifacts affect a larger fraction of the total stream network. Spatially varying tectonics and lithology can have similarly strong influences on optimized values in small catchments for this same reason. In addition, debris flows or other processes important in high-relief terrain may compound these issues at small drainage areas (*42*), which may explain some changes to the “optimized” θ_ref_ values.

### Erosion rates and topography

**Fig. 4.**
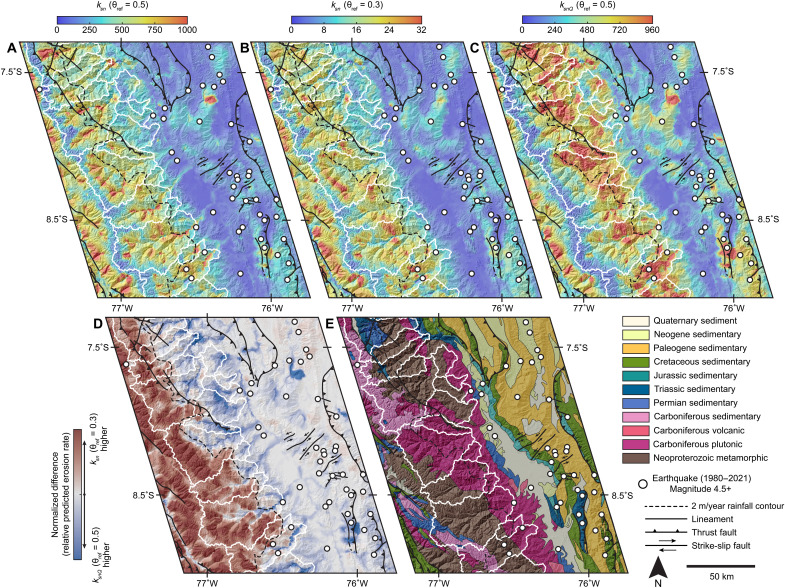
Maps highlighting distinct erosion rate patterns predicted by *k_sn_* and *k_snQ_* and relationships to local geology. Comparison between interpolated maps of *k_sn_* and *k_snQ_* (see Materials and Methods) (**A** to **C**), difference map highlighting contrasts between *k_sn_* and *k_snQ_* (**D**), and simplified geologic map (**E**). Position also shown in figure 1. Color stretches for (A) to (C) are linear minimum-maximum scales cropped to the same data range to be directly comparable while excluding outliers (colors show lower ~99.5% of the full data ranges). Geology and faults were simplified from (*15*), and recent seismicity is from the U.S. Geological Survey earthquake catalog.

Metrics *k_sn_* and *k_snQ_* ultimately predict distinct erosion rate patterns (Figs. 2 and 4). At the broad scale, the *k_sn_* pattern is not sensitive to θ_ref_ and predicts a more homogeneous erosion pattern than *k_snQ_* (Fig. 4, A to C). Also, *k_sn_* systematically predicts higher erosion rates than *k_snQ_* at high elevations because it does not account for the rainfall pattern. Here, the 2 m/year rainfall contour nicely separates domains where *k_sn_* and *k_snQ_* predict higher erosion rates (Fig. 4D). These observations are all consistent with the expectation that many factors are convolved within *k_sn_*. In contrast, to the extent that *k_snQ_* captures the influence of climate, it better isolates tectonic and lithologic factors (Fig. 4, C and E).

**Fig. 5.**
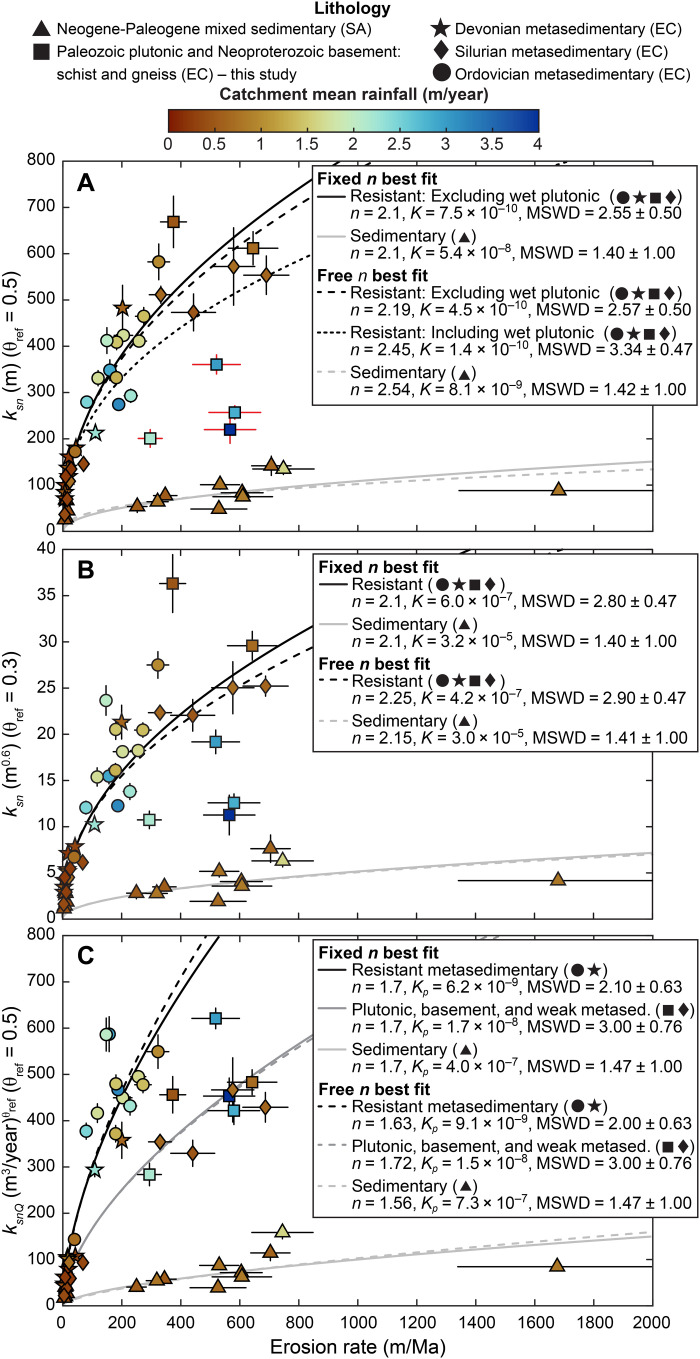
Synthesis of relationships between channel steepness and ^10^Be catchment-average erosion rates (54 in total) from the eastern flank and of north-central Andes. Here, we compile a curated set of published data from northern and central Bolivia (*43*–*46*) and six new rates from southern Peru. EC and SA denote association with the Eastern Cordillera and Subandes, respectively. Measurements are shown with 1σ uncertainty. See Materials and Methods for details about data curation, lithologic classification, and regression analysis. Regressions based on the best-fit fixed *n* values are preferred because they allow direct comparison of *K* (or *K_p_*) values between trends; results from regressions where both *n* and *K* (or *K_p_*) values are allowed to vary are similar and shown for comparison. Samples from catchments with plutonic bedrock and high rainfall (wet plutonic) excluded from fitting in (**A**) are shown with red error bars; however, these samples are not excluded from fitting in (**B**) because θ_ref_ = 0.3 here implies that *K* is independent from rainfall. Note that *k_snQ_* resolves distinct trends among resistant rock types found in the EC that are not evident using *k_sn_*, and best-fit *n* values for *k_sn_* (both θ_ref_) are higher than for *k_snQ_*. Furthermore, note the clustering of plutonic bedrock samples (squares) experiencing wide variations in rainfall in (**C**) compared with scattering exhibited by these same samples in (A) and (B). MSWD, mean square weighted deviation; Ma, million years.

Plotting new and published (*43*–*46*) erosion rates from quasi–steady-state catchments (*E* = *U*) across the study area against *k_sn_* and *k_snQ_* reveals the potential for *k_snQ_* to overcome limitations of *k_sn_* where climate, lithology, and tectonics covary (Fig. 5). Furthermore, consequences of carrying forward faulty assumptions (e.g., quasi-uniform *K* despite variable lithology and rainfall) during topographic analyses are evident. It is difficult to detect any consistent relationships among *k_sn_*, erosion rate, lithology, and climate for catchments in the Eastern Cordillera (EC), particularly for θ_ref_ = 0.3, and only pronounced lithologic contrasts between EC and Subandean catchments are apparent (Fig. 5, A and B). This could be mistakenly interpreted to suggest that the influences from climate in general and lithologic variations in the EC are weak or absent. In contrast, *k_snQ_* resolves distinct relationships separated by threefold differences in *K_p_* between resistant (quartzite-rich) metasedimentary rocks and plutonic/weaker metamorphic rocks comprising EC catchments (Fig. 5C). Clustering, particularly of catchments with plutonic bedrock that experience disparate rainfall but have similar *k_snQ_* values, further supports the interpretation that *k_snQ_* captures the influence of climate. As transient responses to recent changes to the rainfall pattern would tend to disrupt such clustering (*11*), this observation also bolsters the interpretation that catchments reflect quasi-adjustment to the modern rainfall pattern.

This example highlights the need for caution, however, when considering relationships between erosion rates and *k_snQ_*, particularly where strong variations in rainfall are present. Because *k_snQ_* magnifies differences between catchments with different lithology that also experience different amounts of rainfall, it is better able to resolve distinct lithologic trends. However, failure to segregate samples populating these different trends would increase data scattering and result in apparently weaker correlations. Catchments containing more than one lithology are also likely to plot in intermediate positions between distinct endmembers, which can compound apparent scatter and potentially distort endmember trends.

We also find that *k_snQ_* implies that topography is more sensitive to uplift rate than *k_sn_*, reflected by lower values for power-law exponent *n* (Eqs. 2a to 2c) (*2*). While we emphasize that a range of *n* values (and associated erosional efficiency coefficients) can fit these data reasonably well, relationships determined using *k_snQ_* are better constrained than *k_sn_* (see the Supplementary Materials). This apparently weaker topographic sensitivity to uplift rate (higher *n*) and weaker constraints on this sensitivity (wider range of compatible *n* values) are both attributable to the mixing of lithologic and climatic influences in *k_sn_*. In addition, after accounting for rainfall variations with *k_snQ_*, we do not resolve any dependence of *n* on climate, consistent with results from the Himalaya (*1*, *19*). This may reflect continuity of quasi-linear scaling between threshold-exceeding flood discharges and MAP across climate regimes in our study (*1*, *12*). We expect that any climate-dependent departures from this scaling would inhibit data collapse using similar *n* values, particularly among catchments with disparate rainfall. Notably, variable *n* values with climate and breakdown of quasi-linear scaling should also be expressed in collinearity between trunk and tributary rivers, which we do not observe (Fig. 2G). Together, these findings suggest that capturing the influence of climate is an important precondition to understanding interactions among topography, erosion, and other environmental variables.

### Implications for landscape analysis

This analysis reveals important considerations for future landscape analyses. Although we argue that tributaries here have lower *k_sn_* value than trunk rivers due to climate-driven variations in erosional efficiency (*K*), anytime large and small rivers exhibit this pattern, a lower θ_ref_ value will decrease the channel steepness disparity between them. Optimizing collinearity between rivers in χ-*z* space will return the θ_ref_ value that best approximates assumptions required for equal *k_sn_*: spatially uniform *K* and *U*. Under such conditions, differences in slope are directly related to differences in upstream drainage area. In practice, enforcing this condition optimizes the convolution of any environmental variations that exist with drainage area, and systematic spatial gradients in *K* and/or *U* will bias optimized values. This, of course, is problematic if the goal is to extract meaningful information about controls on channel steepness. Furthermore, rather than true variability in θ_ref_, variability in optimized values likely reflects spatially variable *K* and/or *U* (i.e., violations of collinearity requirements, not variations in factors controlling the intrinsic profile concavity represented by *m*/*n* in the SPM). This same reasoning applies to *k_snQ_*. However, unlike *k_sn_* where orographic rainfall can systematically affect *K* between catchments across large areas and effects of these rainfall variations can propagate downstream (e.g., integrating dry headwaters) (*11*), lithology (~*K_p_*) and data resolution limitations primarily affect *k_snQ_* only locally. Collinearity optimization algorithms are better equipped to handle localized perturbations (*36*), decreasing the potential for biased θ_ref_ estimates based on χ*_Q_-z* collinearity.

Despite these strengths, we do not necessarily expect that all landscapes are in quasi-equilibrium with modern MAP. These exceptions may reflect important complexities of the local hydroclimate or recent climate changes that have substantially altered modern rainfall patterns [e.g., (*47*–*49*)]. Also, spatial gradients in uplift can differently influence trunk and tributary *k_snQ_*. In all these cases, optimizing χ*_Q_*-*z* collinearity may also give biased θ_ref_ estimates, the use of which may complicate *k_snQ_*–erosion rate relationships and distort the apparent influence of different environmental variables. Hence, we caution that these factors should be considered when interpreting χ*_Q_*-*z* collinearity.

We conclude that *k_snQ_* based on MAP with θ_ref_ ≈ 0.45 to 0.5 provides the best remote estimate of spatial variations in erosion at the landscape sale. Calibrated with erosion rate measurements, it can markedly improve the understanding of primary environmental controls on landscape evolution. These findings are unaffected by the diverse climatic, tectonic, and morphologic conditions across >1500 km along strike on the eastern flank of the central Andes, demonstrating that climate exerts a strong, ubiquitous influence on erosional efficiency and topography.

## MATERIALS AND METHODS

### Topographic analysis

We use the 1–arc sec (~30-m) Shuttle Radar Topography Mission digital elevation model (*50*) and TRMM-2B31 (Tropical Rainfall Measuring Mission) rainfall data (*51*), which has a ~4.5-km horizontal resolution at this latitude. We extracted and analyzed river profiles from 104 transversely draining catchments (>100 km^2^) that span >1500 km north to south along the eastern margin of the north-central Andes using built-in functions in TopoToolbox and the Topographic Analysis Kit (*52*, *53*). We extract the longest (trunk) river from each of the 104 transverse catchments and all tributaries that drain directly into the trunk stream with drainage area between 5 and 100 km^2^ (1694 in total). Then, we apply the χ-transform (*34*) to stream networks to linearize profiles in χ-elevation plots

$$\chi =\int_{x_{b}}^{x}{\left(\frac{A_{0}}{A(x)}\right)}^{\theta_{\mathrm{ref}}}dx$$ (3a)

$$z(x)=z(x_{b})+k_{sn}\cdot \chi$$ (3b)

where *x* is the distance upstream; *x_b_* is the outlet position; *A*_0_ is a reference drainage area, here set equal to 1 km^2^; and *z* is the elevation. The above equations can similarly be cast in terms of discharge (*Q*) rather than drainage area to arrive at χ*_Q_* and *k_snQ_* (*11*, *40*)

$$\chi_{Q}=\int_{x_{b}}^{x}{\left(\frac{Q_{0}}{Q(x)}\right)}^{\theta_{\mathrm{ref}}}dx$$ (4a)

$$z(x)=z(x_{b})+k_{snQ}\cdot \chi_{Q}$$ (4b)

Collinearity is defined as the degree to which two river segments collapse to a single line in χ-*z* (or χ*_Q_*-z), which we quantify in two ways. The first method directly compares the average channel steepness (*k_sn_* and *k_snQ_*) of tributaries with equivalent segments of its trunk river: Lines may only be collinear if they have equal slopes. We calculate the average channel steepness for each tributary as its fluvial relief divided by the change in χ (or χ*_Q_*), both of which are measured from the tributary confluence with the trunk river upstream to a minimum drainage area of 1 km^2^ (i.e., *k_sn_* = Δ*z*/Δχ). Average *k_sn_* and *k_snQ_* values for comparable segments of the trunk river are calculated using Δχ (or Δχ*_Q_*) along the trunk river over the same elevation range as for each tributary. Results and interpretations are not sensitive to different methods of calculating the average *k_sn_* or *k_snQ_*, for example, with a fixed segment smoothing distance.

The second method optimizes θ_ref_ values for both *k_sn_* and *k_snQ_* that maximize collinearity in χ-*z* and χ*_Q_*-*z*, respectively, in individual catchments using the “mnoptimvar” function in TopoToolbox (*52*) [cf. (*36*, *39*)]. It operates by segmenting stream networks from each catchment into bins of equal χ (or χ*_Q_*). The function then searches for the θ_ref_ value that minimizes elevation differences as quantified by a user-defined variability statistic among stream segments across all bins. Optimization results presented here use the “robustcov” function in MATLAB, which implements an efficient algorithm to estimate the minimum covariance determinant (*54*). Minimizing interquartile range and SD yields comparable results. Results do not appear sensitive to the number of bins or to the minimum number of stream segments within each bin. As discussed above, optimized values reflect those that best align with assumptions for collinearity, which are not necessarily equivalent to the most appropriate θ_ref_ value, particularly in catchments with spatially varying environmental factors (Supplementary Materials).

Interpolated maps for *k_sn_* and *k_snQ_* are all generated from the same stream network. Color ramps for *k_sn_* and *k_snQ_* maps are normalized to reflect equivalent data ranges that include ~99.5% of pixel values. This range was determined to exclude outliers that saturate color maps. The normalized difference map was calculated by first normalizing values in *k_sn_* and *k_snQ_* maps between 0 and 1. Color ramp was then optimized for visualization centered on a value of 0 with symmetrical ranges above and below.

### ^10^Be sample preparation

Southern Peru samples were collected in 2018 from active channel deposits of quasi–steady-state trunk-stream tributaries and processed at the WOMBAT laboratory at the School of Earth and Space Exploration at Arizona State University. Samples were first rinsed and dry-sieved to yield a 250- to 1000-μm fraction. Sieved samples were cleaned using a 2:1 solution of hydrochloric acid (HCl) and nitric acid (HNO_3_). Samples underwent density separation using lithium polytungstate to remove heavy minerals, which was then diluted to separate quartz from less dense minerals. Samples were leached using 1 to 2% hydrofluoric acid (HF) and HNO_3_ solution and rolled on heat for 6 to 8 hours. Samples underwent a minimum of 10 leaches to eliminate mineral species other than quartz and ensure complete etching of quartz grains. Once quartz fractions were purified, samples were spiked with a commercial ^9^Be solution and dissolved in HF and HNO_3_. Beryllium was extracted through standard anion and cation chromatography techniques, oxidized in a muffle furnace, mixed with a niobium matrix, and loaded into cathodes for analysis on the accelerator mass spectrometer at Purdue Rare Isotope Measurement Laboratory. Reported ^10^Be/^9^Be ratios are blank-corrected.

### Erosion rate calculation, curation, and regression analysis

Published erosion rates were recalculated using a common workflow to ensure robust comparisons and curated to exclude catchments unlikely to reflect quasi–steady state conditions. We exclude catchments with large slope-break knickpoints, which unfortunately include a considerable fraction of catchments from the various published datasets. Overwhelmingly, remaining catchments are composed of a single lithology (*15*, *16*), and those comprising more than one lithology are classified according to the dominant lithology by extent within the catchment. Channel steepness for new and published samples in quasi–steady state catchments was calculated with a minimum drainage area of 1 km^2^ and smoothing distances of 500 m. Erosion rates were recalculated using a production-rate weighted elevation following the procedures of Portenga and Bierman (*55*), and published ^10^Be concentrations were scaled to be consistent with 07KNSTD standard (*56*). Erosion rates were then calculated using the CRONUS online calculator [version 3.0 (*57*, *58*)]. We assume no topographic shielding, a sediment thickness of 0 cm, and a density of 2.65 g/cm^3^ and use the “std” elevation flag. Erosion rates are calculated using time-invariant production rate scaling (*59*) and are quoted with 1σ external uncertainty.

Regression analysis was conducted following the methods used by Adams *et al.* (*1*). Quality of fits was evaluated using the mean square weighted deviation, which can account for uncertainties on individual measurements in both *x* and *y* directions. Well-fit models should approach 1 ± 2σ, with higher values indicating that data are overdispersed and the degree to which uncertainties are likely underestimated (*60*). Models are fit using 2σ external uncertainties on erosion rate measurements and 2 SE on *k_sn_* and *k_snQ_*. We fit distinct relationships apparent in *k_sn_*-*E* and *k_snQ_*-*E* in two ways using the “MC York” MATLAB function from the work of Adams *et al.* (*1*). In the first, we allow both the power-law coefficient (*K* or *K_p_*) and exponent (*n*) to vary freely to arrive at the best-fit pair of parameter values. In the second, we fix *n* to find the best-fit *K* or *K_p_* value. These different approaches yield similar parameter values with indistinguishable differences in quality of fits. However, an advantage to fixing the *n* exponent allows for direct comparison of *K* or *K_p_* values between trends and is therefore preferred. Five samples with Neogene-Paleogene age bedrock from Hippe *et al.* (*45*) were excluded from all fitting because they do not correlate with rocks of similar age across the study area, unlike other rock units, and were not fit separately because of their small population and narrow ranges of both channel steepness and erosion rate.

## References

[1] B. A. Adams, K. X. Whipple, A. M. Forte, A. M. Heimsath, K. V. Hodges, Climate controls on erosion in tectonically active landscapes. Sci. Adv. 6, eaaz3166 (2020). 33067243 10.1126/sciadv.aaz3166 PMC7567587

[2] D. Lague, The stream power river incision model: Evidence, theory and beyond. Earth Surf. Process Landforms 39, 38–61 (2014).

[3] K. X. Whipple, R. A. DiBiase, B. T. Crosby, *Bedrock Rivers* (Elsevier, 2013), vol. 9.

[4] D. W. Burbank, A. E. Blythe, J. Putkonen, B. Pratt-Sitaula, E. Gabet, M. Oskin, A. Barros, T. P. Ojha, Decoupling of erosion and precipitation in the Himalayas. Nature 426, 652–655 (2003). 14668861 10.1038/nature02187

[5] V. Godard, D. L. Bourlès, F. Spinabella, D. W. Burbank, B. Bookhagen, G. B. Fisher, A. Moulin, L. Léanni, Dominance of tectonics over climate in himalayan denudation. Geology 42, 243–246 (2014).

[6] H. Seybold, W. R. Berghuijs, J. P. Prancevic, J. W. Kirchner, Global dominance of tectonics over climate in shaping river longitudinal profiles. Nat. Geosci. 14, 503–507 (2021).

[7] D. R. Montgomery, G. Balco, S. D. Willett, Climate, tectonics, and the morphology of the Andes. Geology 29, 579–582 (2001).

[8] S.-A. Chen, K. Michaelides, S. W. D. Grieve, M. B. Singer, Aridity is expressed in river topography globally. Nature 573, 573–577 (2019). 31527826 10.1038/s41586-019-1558-8

[9] D. P. Finlayson, D. R. Montgomery, B. Hallet, Spatial coincidence of rapid inferred erosion with young metamorphic massifs in the Himalayas. Geology 30, 219–222 (2002).

[10] B. Bookhagen, M. R. Strecker, Spatiotemporal trends in erosion rates across a pronounced rainfall gradient: Examples from the southern Central Andes. Earth Planet. Sci. Lett. 327–328, 97–110 (2012).

[11] J. S. Leonard, K. X. Whipple, Influence of spatial rainfall gradients on river longitudinal profiles and the topographic expression of spatially and temporally variable climates in mountain landscapes. J. Geophys. Res. Earth Surf. 126, e2021JF006183 (2021).

[12] M. W. Rossi, K. X. Whipple, E. R. Vivoni, Precipitation and evapotranspiration controls on daily runoff variability in the contiguous United States and Puerto Rico. J. Geophys. Res. Earth Surf. 121, 128–145 (2016).

[13] B. K. Horton, Sedimentary record of Andean mountain building. Earth-Sci. Rev. 178, 279–309 (2018).

[14] B. T. Bishop, S. L. Beck, G. Zandt, L. Wagner, M. Long, S. K. Antonijevic, A. Kumar, H. Tavera, Causes and consequences of flat-slab subduction in southern Peru. Geosphere 13, 1392–1407 (2017).

[15] INGEMMET, “Geological Map of Peru (1:1,000,000)” (Intituto Geologico, Minero Y Metalurgico, 2016).

[16] SERGEOMIN, “Mapa Geologico de Bolivia 1:1,000,000” (La Paz, 2001).

[17] K. X. Whipple, R. A. DiBiase, B. Crosby, J. P. L. Johnson, *Bedrock Rivers* (Elsevier, ed. 2, 2022), vol. 6.

[18] P. Molnar, R. S. Anderson, G. Kier, J. Rose, Relationships among probability distributions of stream discharges in floods, climate, bed load transport, and river incision. J. Geophys. Res. Earth Surf. 111, 10.1029/2005JF000310, (2006).

[19] D. Scherler, R. A. DiBiase, G. B. Fisher, J.-P. Avouac, Testing monsoonal controls on bedrock river incision in the Himalaya and Eastern Tibet with a stochastic-threshold stream power model. J. Geophys. Res. Earth Surf. 122, 1389–1429 (2017).

[20] A. M. Forte, J. S. Leonard, M. W. Rossi, K. X. Whipple, A. M. Heimsath, L. Sukhishvili, T. Godoladze, F. Kardirov, Low variability, snowmelt runoff inhibits coupling of climate, tectonics, and toporgraphy in the Greater Caucasus. Earth Planet. Sci. Lett. 584, 68–70 (2022).

[21] E. Deal, J. Braun, G. Botter, Understanding the role of rainfall and hydrology in determining fluvial erosion efficiency. J. Geophys. Res. Earth Surf. 123, 744–778 (2018).

[22] G. E. Tucker, Drainage basin sensitivity to tectonic and climatic forcing: Implications of a stochastic model for the role of entrainment and erosion thresholds. Earth Surf. Process Landforms 29, 185–205 (2004).

[23] D. Lague, N. Hovius, P. Davy, Discharge, discharge variability, and the bedrock channel profile. J. Geophys. Res. Earth Surf. 110, 10.1029/2004JF000259, (2005).

[24] J. Han, N. M. Gasparini, J. P. L. Johnson, B. P. Murphy, Modeling the influence of rainfall gradients on discharge, bedrock erodibility, and river profile evolution, with application to the Big Island, Hawai'i. J. Geophys. Res. Earth Surf. 119, 1418–1440 (2014).

[25] C. M. Shobe, G. E. Tucker, M. W. Rossi, Variable-threshold behavior in rivers arising from Hillslope-derived blocks. J. Geophys. Res. Earth Surf. 123, 1931–1957 (2018).

[26] B. P. Murphy, J. P. L. Johnson, N. M. Gasparini, L. S. Sklar, Chemical weathering as a mechanism for the climatic control of bedrock river incision. Nature 532, 223–227 (2016). 27075099 10.1038/nature17449

[27] S. M. Olen, B. Bookhagen, M. R. Strecker, Role of climate and vegetation density in modulating denudation rates in the Himalaya. Earth Planet. Sci. Lett. 445, 57–67 (2016).

[28] M. A. Harel, S. M. Mudd, M. Attal, Global analysis of the stream power law parameters based on worldwide ^10^Be denudation rates. Geomorphology 268, 184–196 (2016).

[29] C. Wobus, K. X. Whipple, E. Kirby, N. Snyder, J. Johnson, K. Spyropolou, B. Crosby, D. Sheehan, Tectonics from topography: Procedures, promise, and pitfalls. Spec. Pap. Geol. Soc. Am. 398, 55–74 (2006).

[30] G. E. Tucker, K. X. Whipple, Topographic outcomes predicted by stream erosion models: Sensitivity analysis and intermodel comparison. J. Geophys. Res. Solid Earth. 107, ETG 1-1–ETG 1-16 (2002).

[31] E. Kirby, K. X. Whipple, Expression of active tectonics in erosional landscapes. J. Struct. Geol. 44, 54–75 (2012).

[32] N. M. Gasparini, K. X. Whipple, Diagnosing climatic and tectonic controls on topography: Eastern flank of the northern Bolivian Andes. Lithosphere 6, 230–250 (2014).

[33] J. Han, N. M. Gasparini, J. P. L. Johnson, Measuring the imprint of orographic rainfall gradients on the morphology of steady-state numerical fluvial landscapes. Earth Surf. Process Landforms 40, 1334–1350 (2015).

[34] J. T. Perron, L. Royden, An integral approach to bedrock river profile analysis. Earth Surf. Process. Landforms. 38, 570–576 (2013).

[35] G. H. Roe, D. R. Montgomery, B. Hallet, Effects of orographic precipitation variations on the concavity of steady-state river profiles. Geology 30, 143–146 (2002).

[36] S. M. Mudd, F. J. Clubb, B. Gailleton, M. D. Hurst, How concave are river channels? Earth Surf. Dyn. 6, 505–523 (2018).

[37] B. Gailleton, S. M. Mudd, F. J. Clubb, S. W. D. Grieve, M. D. Hurst, Impact of changing concavity indices on channel steepness and divide migration metrics. J. Geophys. Res. Earth Surf. 126, e2020JF006060 (2021).

[38] L. Goren, M. Fox, S. D. Willett, Tectonics from fluvial topography using formal linear inversion: Theory and applications to the Inyo Mountains, California. J. Geophys. Res. F Earth Surf. 119, 1651–1681 (2014).

[39] S. Hergarten, J. Robl, K. Stuwe, Tectonic geomorphology at small catchment sizes—Extensions of the stream-power approach and the *χ* method. Earth Surf. Dyn. 4, 1–9 (2016).

[40] R. Yang, S. D. Willett, L. Goren, *In situ* low-relief landscape formation as a result of river network disruption. Nature 520, 526–529 (2015). 25903633 10.1038/nature14354

[41] K. Norton, F. Schlunegger, Migrating deformation in the Central Andes from enhanced orographic rainfall. Nat. Commun. 2, 584 (2011). 22158439 10.1038/ncomms1590

[42] J. Stock, W. E. Dietrich, Valley incision by debris flows: Evidence of a topographic signature. Water Resour. Res. 39, 1089 (2003).

[43] E. B. Safran, P. R. Bierman, R. Aalto, T. Dunne, K. X. Whipple, M. Caffee, Erosion rates driven by channel network incision in the Bolivian Andes. Earth Surf. Process Landforms 30, 1007–1024 (2005).

[44] N. Insel, T. A. Ehlers, M. Schaller, J. B. Barnes, S. Tawackoli, C. J. Poulsen, Spatial and temporal variability in denudation across the Bolivian Andes from multiple geochronometers. Geomorphology 122, 65–77 (2010).

[45] K. Hippe, F. Kober, G. Zeilinger, S. Ivy-Ochs, C. Maden, L. Wacker, P. W. Kubik, R. Wieler, Quantifying denudation rates and sediment storage on the eastern Altiplano, Bolivia, using cosmogenic ^10^Be, ^26^Al, and in situ ^14^C. Geomorphology 179, 58–70 (2012).

[46] F. Kober, G. Zeilinger, K. Hippe, O. Marc, T. Lendzioch, R. Grischott, M. Christl, P. W. Kubik, R. Zola, Tectonic and lithological controls on denudation rates in the central Bolivian Andes. Tectonophysics. 657, 230–244 (2015).

[47] J. Galewsky, C. P. Stark, S. Dadson, C.-C. Wu, A. H. Sobel, M.-J. Horng, Tropical cyclone triggering of sediment discharge in Taiwan. J. Geophys. Res. Earth Surf. 111, F03014 (2006).

[48] S. J. Dadson, N. Hovius, H. Chen, W. B. Dade, M.-L. Hsieh, S. D. Willett, J.-C. Hu, M.-J. Horng, M.-C. Chen, C. P. Stark, D. Lague, J.-C. Lin, Links between erosion, runoff variability and seismicity in the Taiwan orogen. Nature 426, 648–651 (2003). 14668860 10.1038/nature02150

[49] E. Gayer, L. Michon, P. Louvat, J. Gaillardet, Storm-induced precipitation variability control of long-term erosion. Earth Planet. Sci. Lett. 517, 61–70 (2019).

[50] T. G. Farr, P. A. Rosen, E. Caro, R. Crippen, R. Duren, S. Hensley, M. Kobrick, M. Paller, E. Rodriguez, L. Roth, D. Seal, S. Shaffer, J. Shimada, J. Umland, M. Werner, M. Oskin, D. Burbank, D. Alsdorf, The Shuttle Radar Topography Mission. Rev. Geophys. 45, RG2004 (2007).

[51] B. Bookhagen, M. R. Strecker, Orographic barriers, high-resolution TRMM rainfall, and relief variations along the eastern Andes. Geophys. Res. Lett. 35, L06403 (2008).

[52] W. Schwanghart, D. Scherler, Short communication: TopoToolbox 2—MATLAB-based software for topographic analysis and modeling in earth surface sciences. Earth Surf. Dyn. 2, 1–7 (2014).

[53] A. M. Forte, K. X. Whipple, Short communication: The Topographic Analysis Kit (TAK) for TopoToolbox. Earth Surf. Dyn. 7, 87–95 (2019).

[54] P. J. Rousseeuw, K. Van Driessen, A fast algorithm for the minimum covariance determinant estimator. Dent. Tech. 41, 212–223 (1999).

[55] E. W. Portenga, P. R. Bierman, Understanding earth’s eroding surface with ^10^Be. GSA Today 21, 4–10 (2011).

[56] A. T. Codilean, H. Munack, T. J. Cohen, W. M. Saktura, A. Gray, S. M. Mudd, OCTOPUS: An open cosmogenic isotope and luminescence database. Earth Syst. Sci. Data 10, 2123–2139 (2018).

[57] G. Balco, J. O. Stone, N. A. Lifton, T. J. Dunai, A complete and easily accessible means of calculating surface exposure ages or erosion rates from ^10^Be and ^26^Al measurements. Quat. Geochronol. 3, 174–195 (2008).

[58] S. M. Marrero, F. M. Phillips, B. Borchers, N. Lifton, R. Aumer, G. Balco, Cosmogenic nuclide systematics and the CRONUScalc program. Quat. Geochronol. 31, 160–187 (2016).

[59] J. O. Stone, Air pressure and cosmogenic isotope production. J. Geophys. Res. Solid Earth 105, 23753–23759 (2000).

[60] I. Wendt, C. Carl, The statistical distribution of the mean squared weighted deviation. Chem. Geol. Isot. Geosci. Sect. 86, 275–285 (1991).

