# Supplementary material for: Isolating climatic, tectonic, and lithologic controls on mountain landscape evolution
Source: Sci Adv. 2023 Jan 20;9(3):eadd8915. doi: 10.1126/sciadv.add8915 (PMC9858509; doi:10.1126/sciadv.add8915)
Supplement: Supplementary file 1 — Figs. S1 and S2 [file sciadv.add8915_sm.pdf]

Science Advances
AAAS

Supplementary Materials for

Isolating climatic, tectonic, and lithologic controls on mountain landscape evolution

Joel S. Leonard *et al*.

Corresponding author: Joel S. Leonard, joel.leonard@asu.edu

Sci. Adv. **9**, eadd8915 (2023)
DOI: 10.1126/sciadv.add8915

The PDF file includes:

Figs. S1 and S2
Legend for data S1

Other Supplementary Material for this manuscript includes the following:

Data S1

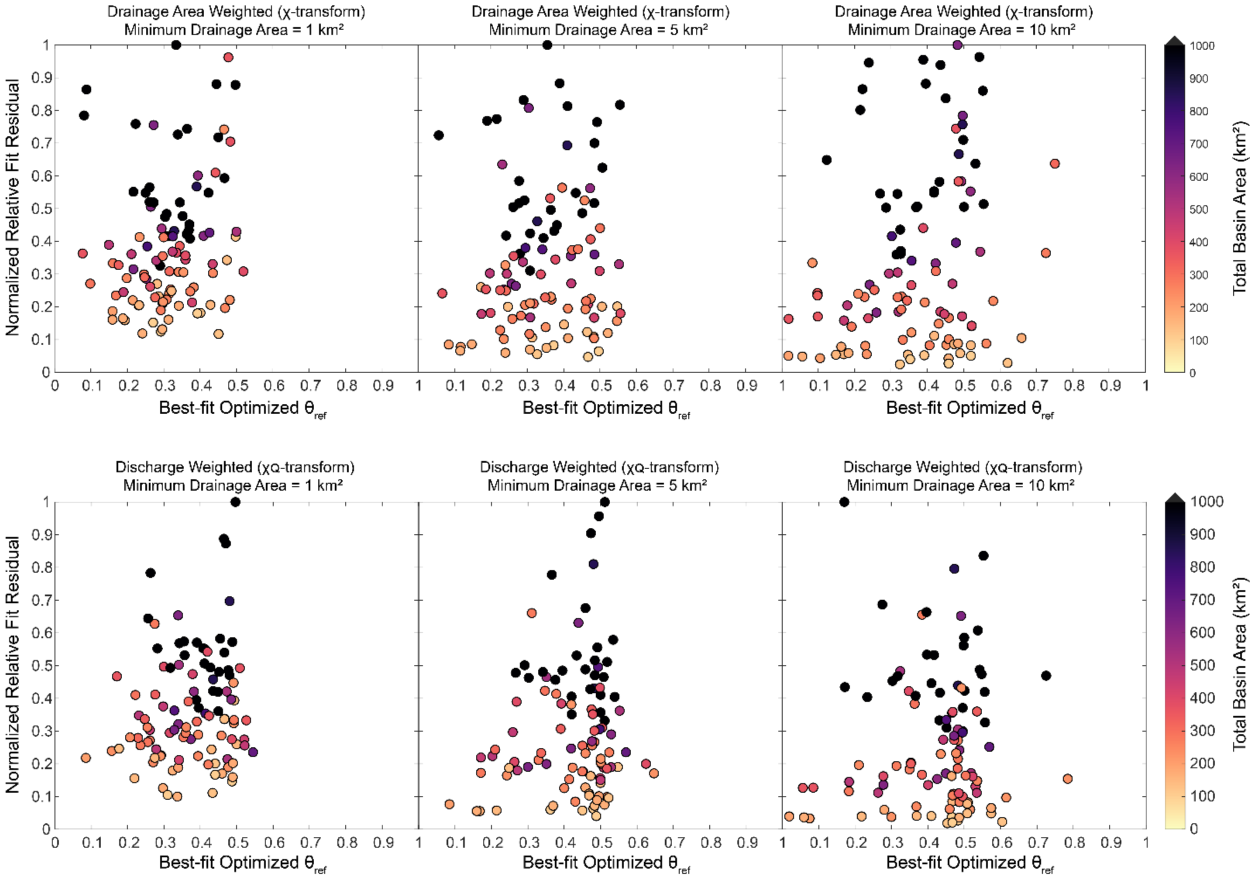

Fig. S1. Comparison between best-fit optimized θ_{ref} and fit residuals for χ-transformed catchments (top) and χ_Q-transformed catchments (bottom), for all minimum drainage area thresholds analyzed (see Figure 3 in main text). Color shows basin size: color stretch restricted to emphasize lower drainage areas. Note, each panel is normalized internally such that residuals for each catchment fall between 0 and 1, and residuals scale roughly with catchment size. Thus, relative characteristics between panels are not necessarily comparable and only comparisons within the same panel among catchments of similar sizes are appropriate. Within each panel, we can observe that catchments with anomalous (low) optimized θ_{ref} exhibit similar residuals to catchments with values near 0.5, demonstrating these anomalous values are indeed effective in collapsing profiles and achieving collinearity, and therefore are not the result of relatively inferior fits. Because these catchments also tend to exhibit variable lithology *(15, 16)* and/or strong disequilibrium, this also demonstrates that adjusting θ_{ref} alone can effectively compensate for and mask meaningful variations in channel steepness.

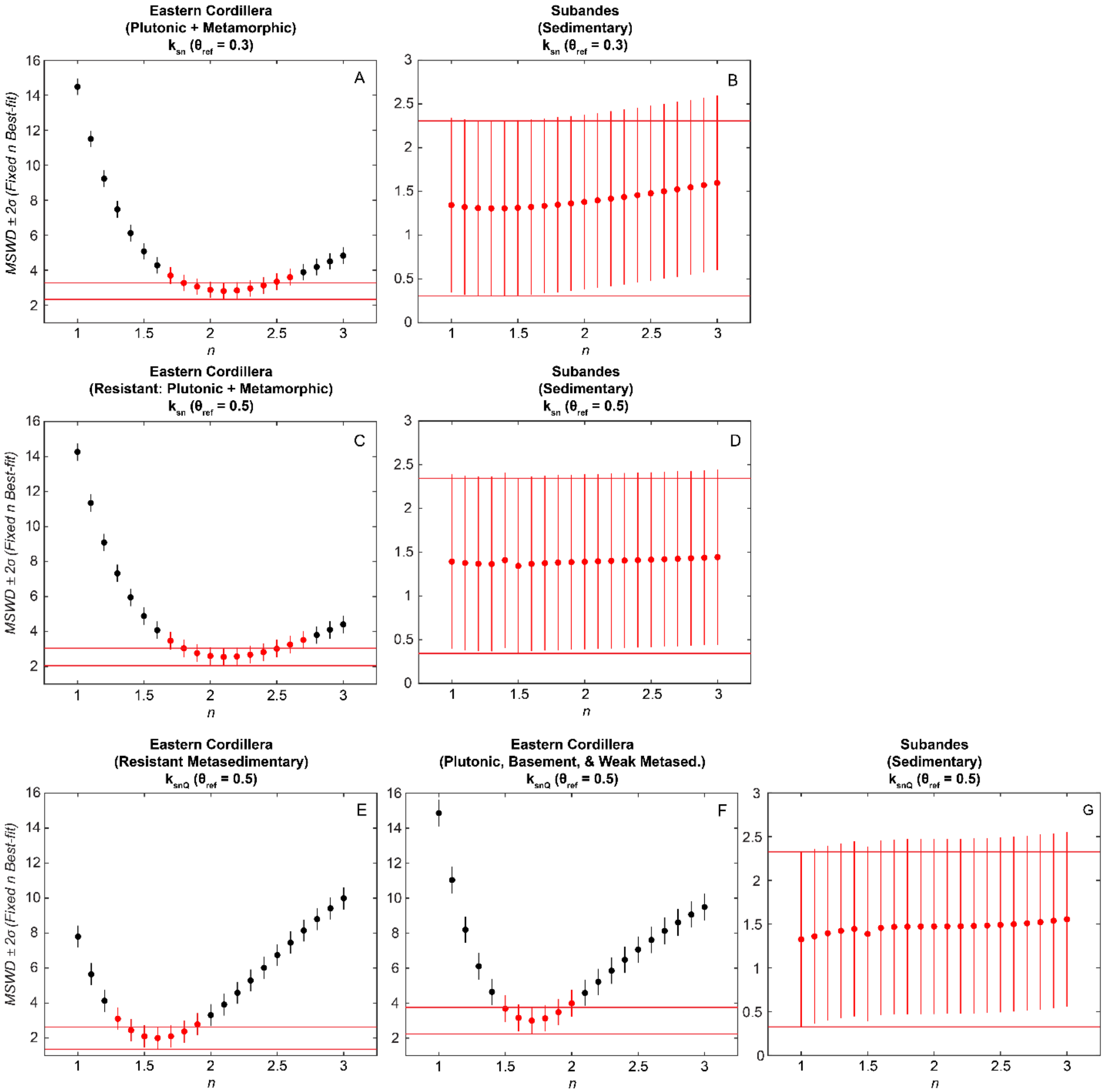

Fig. S2. Constraints on SPM exponent n from regressions for k_{sn} with θ_{ref}= 0.3 (A, B), k_{sn} with θ_{ref} = 0.5 (C, D) and k_{snQ} with θ_{ref}= 0.5 (E-G). Each point records MSWD ± 2σ of best-fit regression (representing a distinct K(or K_p)-n pair) for given fixed n values, calculated over the range of 1-3 at increments of 0.1 using methods from Adams et al. (*1*). Red horizontal lines bound MSWD ± 2σ for the n value of the best-fit among all values (MSWD nearest to 1). Regressions for different n values with MSWD range that overlaps with best-fit n value are shown in red and interpreted to yield comparable goodness-of-fit, thus providing constraints on the range of possible n values. Note, n values for Eastern Cordillera (EC) trends evident using k_{sn} (both θ_{ref}) are higher and less well constrained than either of the EC trends that emerge using k_{snQ}. Subandean trends are unconstrained for both k_{sn} and k_{snQ}. However, assuming an n value for Subandean trends equal to those calculated from EC trends, which are compatible with Subandean trends, implies a K or K_p value that can be directly compared.

Data S1. (separate file)

Source data for all plots and analyses presented in the main text, comprising a spreadsheet with three tabs formatted in excel. Tabs are: 1) 'Large Basin Stats' with topographic characterizations of 104 basins depicted in main text Figs. 1 and 3; 2) 'Trunk-Tributary Pairs' with source data and relevant information for all catchments analyzed to create main text Fig. 2; and 3) 'Erosion Rate Catchment Stats' containing all information to reproduce analysis presented in main text Fig. 5.
